# Could Externalized St. Jude Medical Riata® Lead Be a Culture Medium of a Polymicrobial Endocarditis? A Clinical Case

**DOI:** 10.1155/2017/8967234

**Published:** 2017-01-15

**Authors:** Zefferino Palamà, Roberta Trotta, Cosimo Mandurino, Mariangela Pinto, Giovanni Luzzi, Stefano Favale

**Affiliations:** Cardiology Unit, Cardiothoracic Department, University of Bari, Bari, Italy

## Abstract

We report the case of a man affected by polymicrobial endocarditis developed on a St. Jude Medical Riata lead with a malfunction because of the outsourcing of conductors. The patient was treated with antibiotic targeted therapy and showed different bacteria at the blood cultures and then underwent transvenous leads extraction. Vegetations were highlighted on the caval, atrial, and ventricular tracts of the Riata lead, but the cultures were all negative. The externalization of Riata lead may cause the malfunction but it could also promote bacterial colonies and vegetations. In conclusion, looking for early signs of infection is mandatory during Riata leads follow-up checks.

## 1. Introduction

Cardiac Implantable Electronic Device (CIED) infections rates range between 0,2% and 5,8% [1]. CIED endocarditis accounts for approximately 10% of all cases of device infection [2]. St. Jude Medical Riata ICD leads show an abnormal incidence of insulation defects. The incidence of Riata externalization ranges between 2 and 4% per year. If electrical abnormalities are observed addition of a new lead is recommended; lead extraction is proposed if indicated. Complete removal of all hardware, including leads, is mandatory to ensure eradication of infection as per current guidelines [3].

## 2. Case Report

We report the case of an 83-years-old man admitted in the Infective Disease Department for persistent fever of unknown origin (FUO). The patient was implanted in 2000 with a CRT-D system. Three years later, he presented with a pocket infection and underwent leads extraction. A new CRT-D (St. Jude Medical Atlas® HF V-341) was implanted on the contralateral right side with an atrial lead (Medtronic CapSurefix® Novus 5076) and a right ventricular single coil lead (St. Jude Medical Riata 1572), and a left ventricular lead (St. Jude Medical Quicksite® 1056T).

Ten years later, the patient developed FUO resistant to antibiotics; he was admitted in Infective Disease Department where a transesophageal echocardiography (TEE) showed many vegetations on the leads: the biggest one (2 cm × 3 cm) in the right ventricular lead, near tricuspid valve, without continuity with it. Left ventricular ejection fraction (LVEF) was 55%. Blood tests showed the following: white blood cell 15670/uL (89,9% neutrophils), C-reactive protein 110 mg/l, and procalcitonin 0.9 ng/ml. Different blood cultures were done at admission and were positive to* Staphylococcus epidermidis*. The patient was treated with Daptomycin and Linezolid for 20 days; later on the blood cultures became positive for* Staphylococcus capitis* and then to* Staphylococcus Hominis* subsp.* hominis*, while the patient experienced a progressive decay of the general state, without fever. The TEE, performed about two months later during the therapy, showed reduction of vegetations (the greater one, located on the right ventricular lead, measured 1.1 × 0.9 cm) and a sharp decrease of LVEF (25–30%).

**Figure 1:**
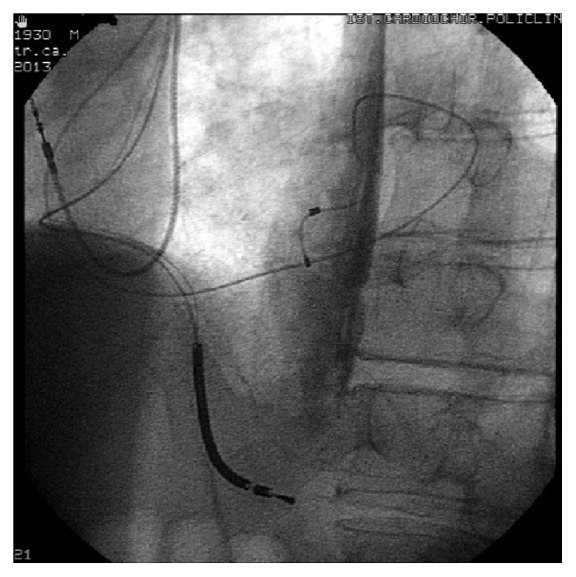
Fluoroscopy after inappropriate shocks.

**Figure 2:**
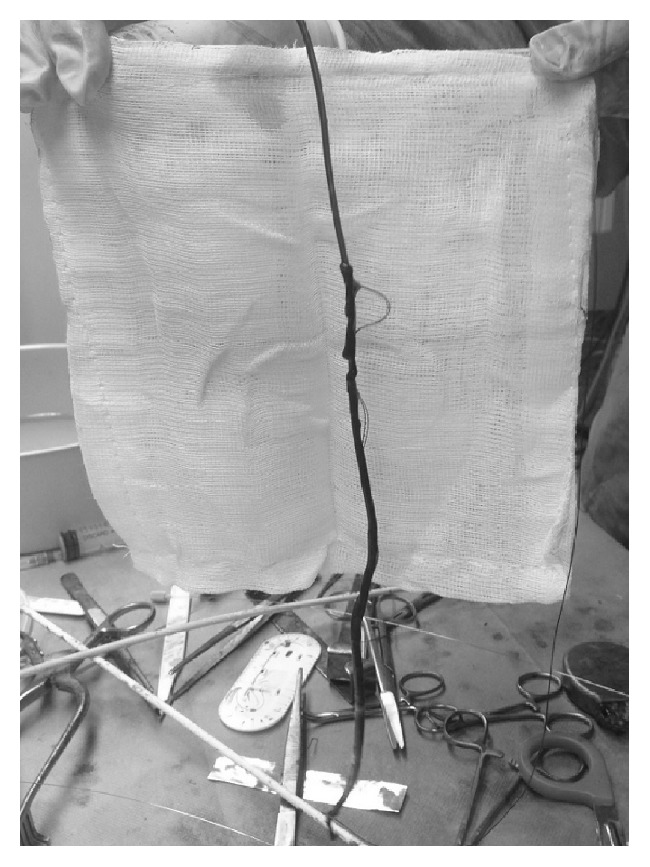
Extracted externalized Riata lead.

The patient was transferred to Cardiology Unit because of two inappropriate ICD's shocks and the evidence of reduction of pacing impedances of RV Riata lead, associated with electrical noise. Fluoroscopy showed the externalization of Riata lead's conductors (Figure 1). The patient underwent transvenous leads extraction. The lead showed three outsourcing instances of conductors in the caval, atrial, and ventricular tracts where numerous adhesions and vegetations were evident (Figure 2). The procedure was successfully completed. The cultures set on the extracted leads were positive to St. hominis. The blood cultures became negative but the patient received antibiotic therapy with Daptomycin and Linezolid for fourteen days because the TEE showed a residual thin floating mass 4 cm in length in the right atrium (ghost). The general state improved and the patient refused ICD reimplantation. At discharge blood tests showed the following: white blood cell 5530/uL (64,4% neutrophils), C-reactive protein 8.5 mg/l, and procalcitonin < 0,1 ng/ml. Currently the patient is regularly followed up in our electrophysiology outpatient clinic. Two months later, a transthoracic echocardiography confirmed the thin atrial mass (ghost) and an improvement of LVEF (35%).

## 3. Discussion

Externalized Riata lead is a known problem and this case is an example of CIED's transvenous extraction because of an endocarditis with a supervened lead's electrical malfunction. The endothelial lesions and alterations of flow near an externalized lead represent the prime mover for the expression of prothrombotic factors and the formation of thrombi [4]. Blood clots can be a culture medium for bacterial species that are found during transient bacteremia. Alteration of flow due to an externalized Riata could have represented the starting point for the endocarditis. The continuous expression of adhesion factors close to an externalized lead may have determined that different bacterial species, during transient bacteremias, have been multiplied resulting in polymicrobial endocarditis. The prolonged septic state, through the expression of proinflammatory factors, may have been responsible for the marked decline in LVEF [5]. The endocarditis could have been a consequence of the lead's outsourcing; the lead dysfunction appeared later. CIED's transvenous extraction has allowed a rapid improvement in the clinical state.

## 4. Conclusion

Externalized Riata lead is a known problem. This clinical case is an example of an endocarditis most likely resulting from outsourcing of the lead. The quarterly or half yearly monitoring of electrical parameters of these leads should be accompanied by a continuous clinical monitoring of the patient in order to identify early signs of infection suspected for endocarditis.
